# Acfs: accurate circRNA identification and quantification from RNA-Seq data

**DOI:** 10.1038/srep38820

**Published:** 2016-12-08

**Authors:** Xintian You, Tim OF Conrad

**Affiliations:** 1 Department of Numerical Mathematics, The Zuse Institute Berlin, Berlin, 14195, Germany; 2 Institut für Mathematik, Freie Universität Berlin, Berlin, 14195, Germany

## Abstract

Circular RNAs (circRNAs) are a group of single-stranded RNAs in closed circular form. They are splicing-generated, widely expressed in various tissues and have functional implications in development and diseases. To facilitate genome-wide characterization of circRNAs using RNA-Seq data, we present a freely available software package named acfs. Acfs allows *de novo*, accurate and fast identification and abundance quantification of circRNAs from single- and paired-ended RNA-Seq data. On simulated datasets, acfs achieved the highest F1 accuracy and lowest false discovery rate among current state-of-the-art tools. On real-world datasets, acfs efficiently identified more *bona fide* circRNAs. Furthermore, we demonstrated the power of circRNA analysis on two leukemia datasets. We identified a set of circRNAs that are differentially expressed between AML and APL samples, which might shed light on the potential molecular classification of complex diseases using circRNA profiles. Moreover, chromosomal translocation, as manifested in numerous diseases, could produce not only fusion transcripts but also fusion circRNAs of clinical relevance. Featured with high accuracy, low FDR and the ability to identify fusion circRNAs, we believe that acfs is well suited for a wide spectrum of applications in characterizing the landscape of circRNAs from non-model organisms to cancer biology.

CircRNAs were discovered over two decades ago as a special group of RNA transcripts featuring circular structures^1^,^2^,^3^,^4^,^5^. Recent advancements in high-throughput sequencing technologies and experimental protocols enable unbiased deep profiling of circRNA landscape in a genome-wide manner, leading to the re-discovery of thousands of circRNAs in eukaryotes^6^,^7^,^8^,^9^ and archaea^10^. CircRNAs are widely expressed and regulated in organisms such as human, mouse, rat, fruit fly and *C. elegans*^11^,^12^,^13^,^14^,^15^. CircRNAs are generated through alternative splicing, where a downstream splice donor is covalently linked to an upstream splice acceptor, forming a characteristic back-splice junction(BSJ) (Fig. 1a). CircRNAs can originate from multi-exonic transcripts, single exonic transcripts, uncharacterized transcripts and even fusion genes^16^. Alternative RNA processing events have been observed in circRNAs, including exon skipping, intron retention and alternative splicing^13^,^17^,^18^. The vast isoform diversity, tight regulation of expression and deep evolutionary conservation collectively suggest the potential functionalities of circRNAs. It has been proposed that circRNAs could exert their functions by several means including decoying miRNA and RNA binding proteins (RPBs)^19^,^20^. Although the detailed mechanisms are still under scrutiny, dysregulation of circRNAs has been linked to diseases such as Alzheimer and Leukemia^16^,^21^. Moreover, due to the exceptional biochemical stability endowed by their circular form, circulating circRNAs are also enriched in exosomes and can therefore serve as a promising biomarker for cancer diagnosis^22^.

It is critical to accurately identify and quantify circRNAs in samples of interest so that we can gain more insights of the expression dynamics and biological functions of circRNAs. Many methods have been developed to detect circRNAs using RNA-Seq data (Table 1), yet there are still five challenges to be addressed. Firstly, the characteristic BSJs should be identified genome-wide in an unbiased manner. Several tools, such as CIRCexplorer^23^, KNIFE^24^ and MapSplice2^25^, rely on a priori gene annotation and only check for possible BSJs consisting of known exons(Table 1). Despite the advantage of these reference-guided methods in which the search space is greatly reduced from the genome to annotated exons, they are not able to detect circRNAs that contain unannotated exons or splicing sites or those originated from unannotated gene loci. For example, the splice sites of the well-known circRNAs circSRY^2^ and circCDR1as^26^ are not annotated in linear RNA transcripts, and therefore these circRNAs will not be reported by reference-guided methods. Secondly, the authenticity of predicted BSJs should be scrutinized. Several tools, including CIRCexplorer^23^, circRNA_Finder^27^, CIRI^28^, and find_circ^11^, report a candidate BSJ if a canonical splicing motif, such as “GU-AG”, is found(Table 1). Consequently, many *bona fide* splice sites of non-canonical dinucleotide composition^29^ will be discarded. Moreover, splicing sites are not determined solely by these four bases, but much longer sequences^30^. Given the complexity of splicing signals, it is clear that circRNA discovery will benefit from a comprehensive BSJ identification algorithm. Thirdly, accurate abundance quantitation holds the prerequisite of detailed study of regulation and potential functions of circRNAs. Although circRNA abundance could be measured by counting the number of reads consistent with BSJ, many *bona fide* BSJ reads might not be identified during the initial alignment due to the restrictions of the read aligners. As a remedy, realignment to predicted BSJ references can help to improve the quantification accuracy. Furthermore, several state-of-the-art tools only accept paired-end reads as input, which severely restrict their applications (Table 1). Lastly and importantly, none of the current tools allows detection of fusion circRNAs, which is shown to have a role in diseases such as rendering drug-resistance in leukemia^16^.

To address all the aforementioned challenges, we present acfs (accurate circRNA finder suite) for *de novo* circRNA identification and quantification. We showed that acfs is highly accurate, has very low FDR, and can handle both single-end (SE) and paired-end (PE) data. Using a set of simulated datasets and two additional published datasets, we showed that acfs exhibit the best performance comparing to other state-of-the-art tools. Moreover, among thousands of circRNAs identified in leukemia samples^16^,^31^, a subset of them showed distinctive expression pattern and could serve as diagnostic biomarkers. We believe that accurate identification and expression quantitation of circRNAs, as enabled by acfs, will shed light on further understanding of the biogenesis, regulation, and functions of circRNAs.

## Results

### CircRNA identification in acfs

**Figure 1:**
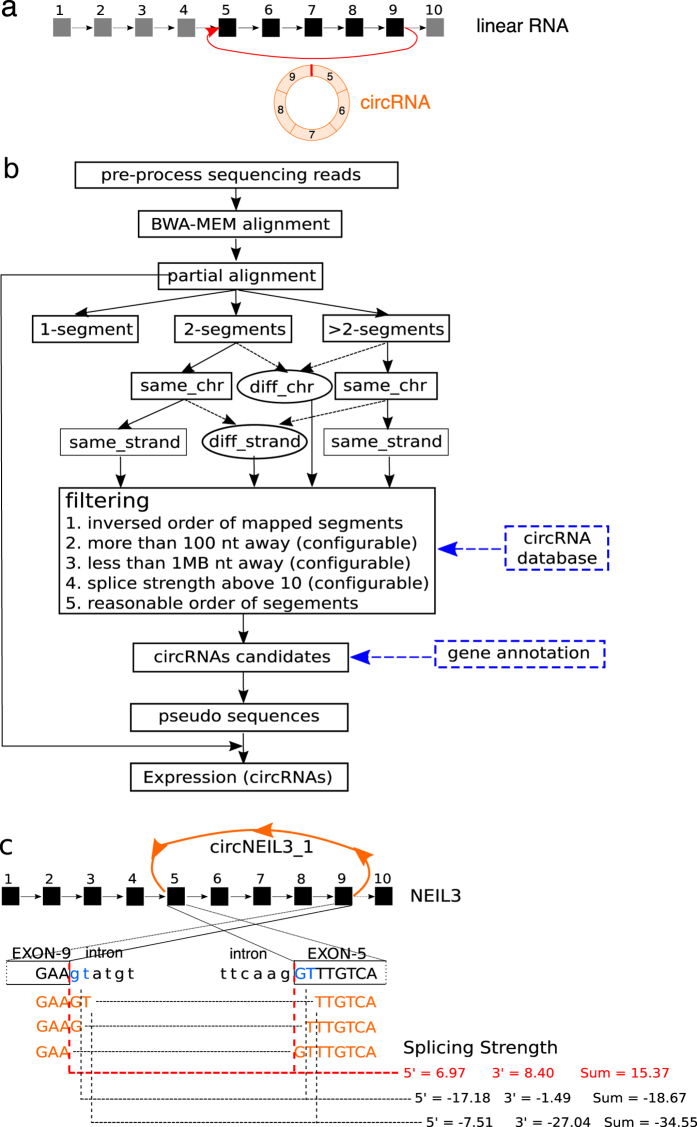
Computational pipeline of circRNA profiling. (**a**) An example of a circRNA (colored in orange) originated from a multi-exonic gene locus (black squares). The red vertical bar on the circRNA marks the BSJ. (**b**) Schematic workflow of acfs, ovals denote processes for fusion circRNA identification, dashed boxes denote optional input. (**c**) Determination of the BSJ using circ_NEIL3 as an example. The BSJ supporting read can be partitioned in three manners (colored in orange) due to the sequence similarity at the splice sites. Exon sequences are shown in upper case, and intron sequences are shown in lower case. Splice strength is estimated for each of the potential splice sites using the maxEnt model. One partition pattern (red dash-line) is predicted to be generated by the splicing machinery and is reported as the BSJ.

Here, we briefly describe the analysis workflow of acfs. As illustrated in Fig. 1b, acfs consists of three main steps: preprocessing, identification and quantification.

### Preprocessing

SE RNA-Seq reads are collapsed, indexed and then aligned to the reference genome sequences using the split-read mapper BWA-MEM^32^. Although acfs is designed to pinpoint the BSJs using SE RNA-Seq reads, conversion of PE data to SE data allows acfs to process PE data as well (termed as “acfs_p” mode). A read pair could be treated either as two independent SE reads if the two mates do not overlap with each other, or merged into one read using the overlapped sequence (see Supplementary Materials). This PE to SE conversion also ensures each read or read-pair is counted only once in the quantification step.

### Identification

Reads potentially originated from the BSJs are examined by first selecting those whose aligned segments locate on the same chromosome and the same strand. Afterwards, acfs examines the splice strength of each candidate BSJ using maxEnt model^30^ and pinpoint the exact genomic position if it meets all the criteria. We illustrate this in an example (Fig. 1c). Here, the circRNA originates from NEIL3 gene and consists of exon5 to exon9, in which the 3′ end of exon9 is covalently linked to the 5′ end of exon5 thereby forming a circular structure (orange arc). A circRNA supporting read (orange sequence) could be partitioned in three equally good manners due to the sequence similarity at the splice sites (colored in blue). However, this ambiguity can be resolved by inspecting the splicing strength of each of the potential splice patterns. The pattern predicted with the highest splice strength (red dashed-lines) passes the threshold and is therefore classified as a BSJ while the other two are unlikely to be generated by splicing. The threshold is user-definable, and we set it to 10 by default to allow correct identification of over 95% of all canonical splicing sites in human transcriptome (Fig. S1). Similarly, reads with segments locate on different chromosomes and/or strands can be examined for fusion junctions of fusion circRNAs.

### Quantification

Acfs deploys an additional alignment procedure to accurately assess the abundance of the predicted circRNAs. Reference sequences of circRNAs are obtained by extracting the sequence between the BSJs, or concatenating the internal exonic sequences if gene annotation is provided. In order to allow aligning reads directly to the BSJs, acfs repeats the reference sequence once for each circRNA and thus generates a pseudo circular reference. Then, acfs aligns all reads to this pseudo circular reference and inspects alignments spanning the BSJs before reporting the circRNA abundance.

To sum up, the major advantages of acfs lie in facts that it allows an unbiased back-splice sites identification without relying on available gene annotation, it inspects the strength of potential splicing sites including but not limited to the canonical “GU-AG” motif, and the additional realignment allows better expression estimation.

### Simulation benchmarks

We designed a set of simulation studies to compare the performance of acfs with seven state-of-the-art tools listed in Table 1. To allow a fair comparison between *de novo* tools and reference-guided tools, we simulated circRNAs only from annotated exons of human RefSeq genes. To recapitulate the diverse splicing events of circRNAs observed in real datasets, we also simulated two circRNAs for a fraction of RefSeq genes. In total, we simulated 61488 circRNAs from RefSeq transcripts. For positive datasets (circRNA reads), we randomly sampled SE or PE reads spanning the BSJ with different read lengths (50, 100, or 150 nt respectively). Similarly, we sampled reads from RefSeq transcripts for negative datasets (linear RNA reads). To rule out the possible influence of transcript abundance, we sampled 20 reads from every circRNA or RefSeq transcript. To further mitigate the potential bias of the performance of different read mappers in use, we kept the simulated reads error-free. Details on the simulation procedures are described in the Methods section.

**Figure 2:**
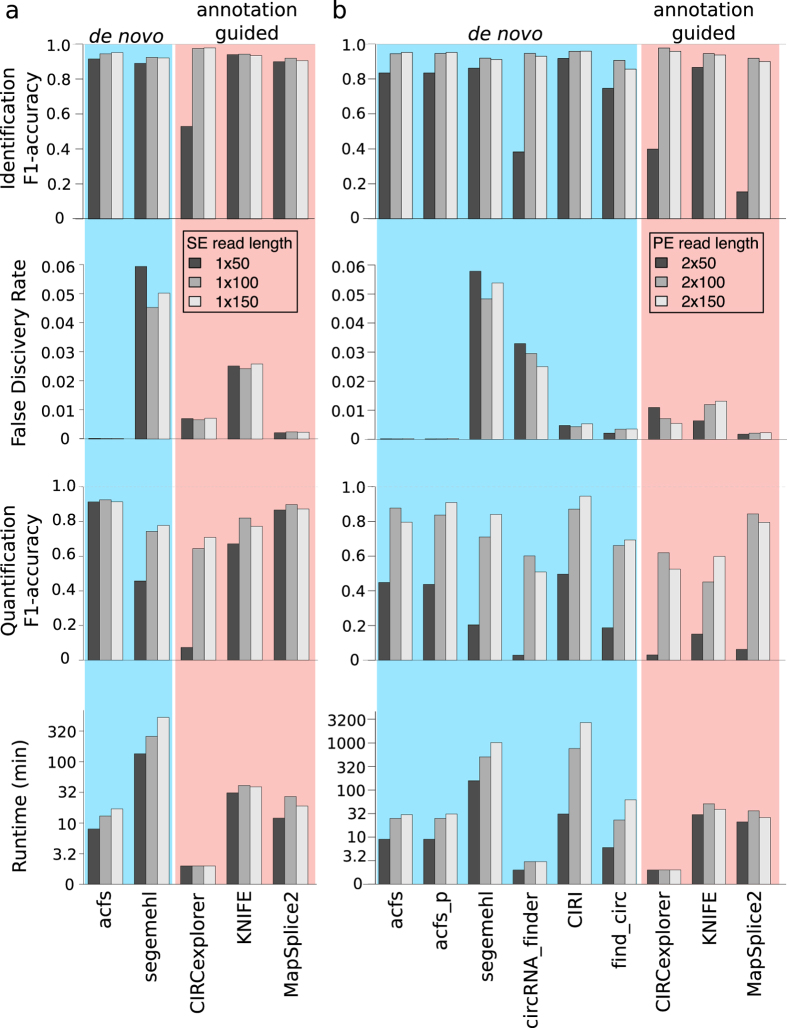
Benchmark of acfs using simulated data emphasizes the importance of accurate BSJ identification. (**a**) Benchmark of all five tools supporting SE datasets in terms of identification accuracy, false discovery rate, quantification accuracy and runtime. (**b**) Benchmark of acfs with seven state-of-the-art tools on PE datasets. The color of the bars (black, grey and white) represents reads of different length (50, 100, 150 nt).

Using the simulated datasets, we benchmarked the performance of acfs together with other state-of-the-art tools listed in Table 1. On simulated SE datasets (Fig. 2a, Table S1), although all tools exhibited high F1 accuracy in detecting circRNA, acfs consistently reported the lowest false positive rate (FDR) that is one to two orders of magnitude smaller than the other tools. Importantly, acfs demonstrated the highest F1 accuracy in estimating the abundance of circRNAs, which is crucial for further analysis of the regulation of circRNAs and highlights the efficacy of the realignment procedure. On simulated PE datasets (Fig. 2b, Table S1), all tools exhibited similar high F1 accuracy in predicting circRNAs when using datasets of longer read length, yet several showed a drastic decrease with shorter reads. Similar to that in SE datasets, the FDR of acfs was orders of magnitude lower than all other tools. Acfs had the second highest F1 accuracy in abundance quantification, tightly following CIRI. In terms of speed performance, acfs ranked the first except for CIRCexplorer and circRNA_finder, both of which use STAR as the read mapper. This gain of speed powered by STAR might in turn be responsible for the high FDR and low F1 accuracy in abundance quantification. Together, acfs achieved a balanced performance in speed and accuracy. Interestingly, although all circRNAs were simulated from RefSeq transcripts, none of the annotation guided tools reported all of them. This upper limit could be partly explained by the presence of pseudogenes or genes belonging to the same family that share highly similar sequences. Of note, since we only simulated circRNAs from annotated transcripts, we systematically underestimate the performance for *de novo* tools. In real-life studies, thousands of circRNAs have been identified with unannotated exon borders or from unannotated gene loci which underlines the efficacy of *de novo* circRNA identification.

### Application on real datasets

**Figure 3:**
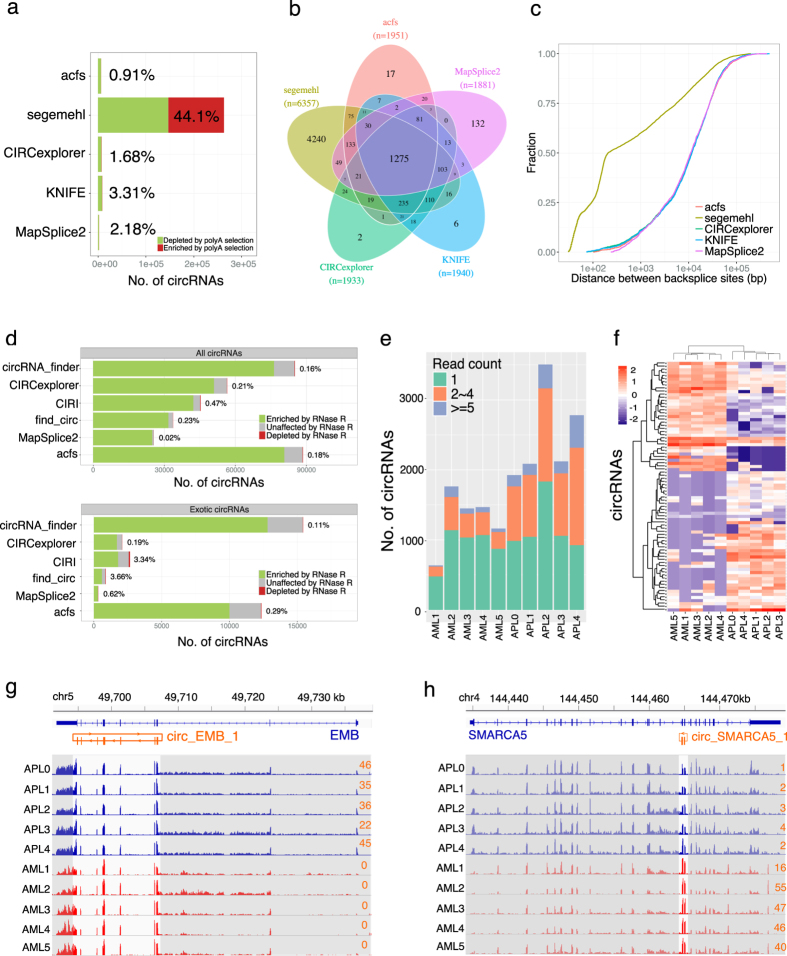
Application of acfs on real datasets suggests the high performance of acfs. (**a**) Barplot shows the number of circRNAs identified by acfs(6363), KNIFE(8774), CIRCexplorer(7254), segemehl(243004) and Mapsplice2(1923) in Dataset A, and acfs shows the smallest FDR. (**b**) Venn diagram of the overlap of circRNAs (> = 2 reads) among five circRNA prediction tools in Dataset A. (**c**) Cumulative distribution of the splice distance for circRNAs predicted in Dataset A. (**d**) Acfs identified more RNase R resistant circRNAs than five other tools on Dataset B with low FDR. (**e**) The number of circRNAs identified in the APL and CN-AML samples with different thresholds. (**f**) Heatmap of 80 differentially represented circRNAs between CN-AMP and APL samples. (**g,h**) Representative examples of circRNAs that are enriched in APL (**g**) and CN-AML (**h**). Light-colored regions highlight the read coverage in the circRNAs. The numbers (colored in orange) to the right of both panels (**g,h**) denote the number of reads supporting the BSJ in each sample.

After demonstrating the high performance of acfs with simulated datasets, we further benchmarked it with two published datasets. The first dataset (dataset A) consists of two SE sequencing libraries^13^: one for total RNA and the other for polyA-selected RNA from mouse brain tissues. Since circRNAs do not process polyA tails and have a low chance of containing a stretch of genomic As in their sequences (Fig. S2), they would be expected to be depleted in the polyA-selected library. We applied all five tools that can process SE data to this dataset. Indeed, the majority of circRNAs detected by all four tools except for segemehl were depleted in the polyA-selected library, and acfs had the lowest FDR (Fig. 3a). Out of 6683 circRNAs predicted by five tools with at least two reads spanning the BSJs, 1275 (19%) were identified by all five tools (Fig. 3b). This low commonality could be attributed to the fact that the majority (66%) of the circRNAs identified by segemehl were not reported by any other tool. To assess the authenticity of these segemehl-specific circRNA candidates, we went on to examine their genomic distance within the predicted BSJs, as suggested by Hansen *et al*.^33^. Whilst circRNAs reported by acfs, CIRCexplorer, KNIFE and MapSplice2 showed a similar distance distribution within BSJs, over half of the segemehl-specific circRNAs were identified with proximal splice sites (<500 nt) and 25% were even within 100 nt (Fig. 3c and Fig. S3). Considering the high proportion of circRNAs that was not depleted by polyA-selection and also the high proportion of their proximal splice sites, many circRNAs predicted by segemehl are more likely to represent artifacts. This observation emphasizes the importance of splice site examination for circRNA identification. Moreover, there were 92 circRNAs identified by acfs that were not reported by tools relying on gene annotation, and 62 of them were originated from unannotated gene loci.

The second dataset (dataset B) consists of four PE deep sequencing libraries from Hs68 fibroblast cells^8^: two replicates from RNase R treated RNA and the other two from untreated RNA. Due to their closed circular structure, circRNAs have much higher resistance to exoribonuclease, such as RNase R, comparing to linear RNA transcripts and are therefore expected to be enriched in the RNase R treated RNA libraries. We applied acfs on this dataset and compared with the results adopted from Hansen *et al*.^33^. In order to get a full landscape of identified circRNAs in this dataset, we considered all circRNA with at least one read spanning the predicted BSJ. Acfs reported the highest number of circRNAs (Fig. 3d). Similar to the other tools, most circRNAs identified by acfs were enriched in the RNase R treated RNA libraries, indicating the authenticity of the predicted circRNAs. We observed that acfs and circRNA_finder were more sensitive in circRNA identification than the other tools. Whilst acfs predicted more circRNAs than circRNA_finder, the circRNAs reported only by acfs is fewer than that of circRNA_finder (Fig. 3d), suggesting circRNAs identified by acfs were better supported by other tools and therefore more likely to be authentic. Furthermore, *de novo* tools reported more circRNAs than those relying on existing gene annotations, emphasizing their utility to profile circRNAs unbiasedly. We also found a subset of circRNAs of high abundance in the untreated RNA library whose representation was drastically depleted in the RNase R treated RNA library. Among these RNase R sensitive circRNAs, many are well known, including circ_CDR1as, which is over 10-fold depleted by RNase R treatment. Although experimental validation of the circular structure of these RNase R sensitive circRNAs *in situ* remains challenging, Hansen *et al*.^26^ suggested that they might represent miRNA-directed AGO2-cleavage products. To examine the generality of the miRNA-guided linearization phenomenon, we predicted for all circRNAs the potential cleavage sites (see Methods). Indeed, we observed a statistically significant negative correlation between the circRNA-miRNA binding affinity and the resistance to RNase R (Fig. S4a). In addition, the secondary structure might also influence the stability of circRNAs when challenged with RNase R, as those with more complex structures show stronger RNase R resistance (Fig. S4b). Taken together, acfs accurately identifies circRNAs with low FDR on both SE and PE RNA-Seq data.

### CircRNAs as biomarkers distinguishing APL and CN-AML samples

To illustrate the potential utility of acfs in biomedical research, we investigated the circRNA landscape in leukemia samples. Acute Promyelocytic Leukemia (APL) is a subtype of Acute Myeloid Leukemia (AML) with the characteristic chromosomal translocation involving retinoic acid receptor alpha (RARA) gene and Promyelocytic leukemia protein (PML) gene. The fusion protein PML-RARA underlines the etiology of this diseases and also points to the high efficacy of the corresponding treatment^34^. However, no obvious genomic alterations could be detected by conventional band analysis in about 40% of all AML cases, and these cases are therefore termed as cytogenetically normal (CN-AML)^31^. We then asked whether circRNA profiles could be used for molecular disease stratification and we identified thousands of circRNAs, using acfs, in five APL samples^16^ and five CN-AML samples^31^. Due to the relatively low sequencing depth, about half of the circRNAs were detected with only one read and a fraction of them had at least five reads (Fig. 3e). Although circRNAs were generally of lower abundance, as a group, they could separate APL from CN-AML samples better than linear protein-coding genes (Fig. S5a,b). Since these two datasets were generated in two different labs, to mitigate the potential influence of batch effects, we used the relative contribution of circRNAs as a proxy of their absolute abundance. The relative contribution (RC) of a circRNA is the quotient of its estimated abundance over that of its hosting gene. The rationale is that the potential bias introduced during the library preparation and sequencing should influence the representation of circRNA and its hosting gene similarly, and using the quotient of the two could remove the bias and thus enable cross-sample comparison. We found 80 circRNAs demonstrating opposite pattern of expression in CN-AML versus APL, which might serve as a set of biomarkers to distinguish the two types of diseases (Fig. 3f, S5c). Interestingly, many circRNA hosting genes are functionally crucial to the differentiation and proliferation of myeloid cells and therefore likely underline the pathogenesis of leukemia. For example, circ_EMB is highly abundant in APL but not in AML samples (Fig. 3g). EMB (Embigin) encodes a transmembrane protein belonging to the immunoglobulin superfamily that has been identified as a biomarker for cancer progression^35^. In addition to the potential pathogenic role of circ_RMB, the conversion of linear EMB transcript to circRNAs via back-splicing might also serve to decrease the protein output and thereby lead to excessive cell proliferation. On the other end of the spectrum, the circ_SMARCA5 is highly abundant in the AML but almost absent in APL samples (Fig. 3h). SMARCA5 (SWI/SNF-Related Matrix-Associated Actin-Dependent Regulator of Chromatin A5) encodes a core component of chromatin remodeling and spacing factor RSF, which promotes cell proliferation^36^ and is dysregulated in primitive hematopoietic cells of AML^37^. We found a miR-10b (a member of the miR-99 family) binding site on circ_SMARCA5, and the miR-99 family has been shown to modulate the expression of SMARCA5^38^. Therefore, we speculated that circ_SMARCA5 could function as a decoy of miR-10b, hence relieve linear SMARCA5 transcripts from repression and eventually contribute to the accumulation of undifferentiated myeloid cells. Although the detailed mechanism in which circRNAs are regulated and function remain for further experimental investigation, we observed that the gene loci frequently mutated in AML cohorts^39^ produced circRNAs with significantly higher abundance, suggesting an alternative approach of gene dysregulation deployed by cancer cells to gain and remain selective advantages against the immune surveillance (Fig. S6).

### Fusion transcript derived circRNAs

**Figure 4:**
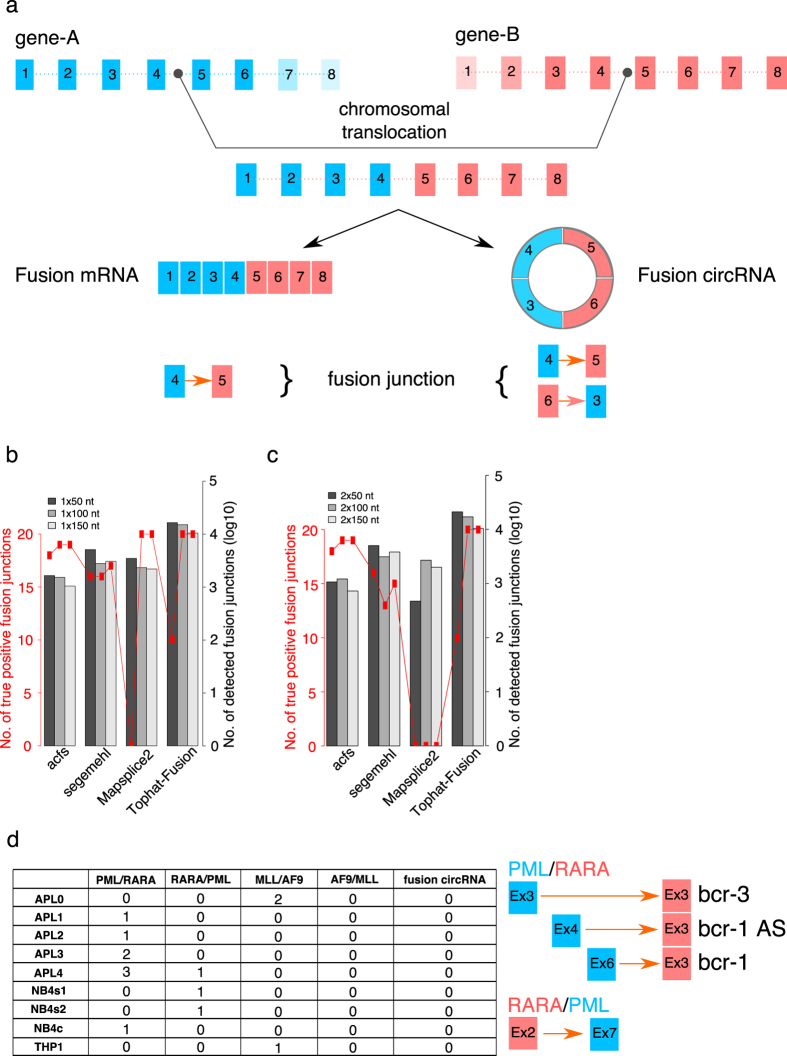
Fusion circRNAs identification by acfs. (**a**) Schematic view of fusion circRNA biogenesis and in silico identification. (**b,c**) Benchmark of acfs with other state-of-the-art fusion prediction tools using simulated SE (**b**) and PE (**c**) datasets. (**d**) Reanalysis of a published datasets detected the characteristic fusion junctions but no fusion circRNAs.

Apart from detecting circRNAs originated from genes in canonical configuration, here we further demonstrated the power of acfs in identifying circRNAs from non-canonical gene structure, such as fusion genes. Chromosomal translocation is observed in numerous diseases, and many fusion genes have been causally linked to tumor development^40^,^41^. Since circRNAs are generated by the splicing machinery, it would be expected that fusion gene loci have also the potential for circRNA biogenesis. A recent study experimentally demonstrated both the existence and the functionality of fusion circRNAs in cancer^16^. However, comprehensive identification of fusion circRNAs in a genome-wide manner remains yet challenging due to the lack of appropriate tools. Although many tools have been developed to detect fusion events, such as MapSplice, Tophat-Fusion^42^ and STAR-Fusion^43^, none could directly report fusion circRNAs. In this study, we showed that acfs could facilitate the profiling of fusion circRNAs using RNA-Seq data. As depicted in Fig. 4a, a fusion mRNA transcript harbors one fusion junction, whereas a fusion circRNA harbors two in a specific order. By determining the two characteristic fusion junctions between two gene loci, fusion circRNAs can be identified. To test the performance of acfs in fusion circRNA identification, we first simulated ten fusion circRNAs, randomly extracted ten reads from each of the fusion junctions, merged them with the simulated dataset described before and benchmarked acfs with other state-of-the-art fusion detection tools. Across SE datasets with different read lengths, acfs consistently identified over 90% of all fusion junctions, whereas MapSplice2 and Tophat-Fusion had marked decrease in reporting true positives on the dataset with shortest read length, and performance of segemehl was consistently inferior to that of acfs (Fig. 4b). We observed a similar trend on the benchmark using PE data, except that MapSplices2 reported none of the true positives for all three datasets (Fig. 4c). Tophat-Fusion identified all fusion junctions for longer read datasets, which came along with the cost of 10-fold more false positives than that of acfs(Fig. 4b,c). The reason behind the fusion junctions missed by acfs was the existence of gene family members with high sequence similarity, which is challenging to resolve using only short-read NGS data. After demonstrating the efficacy in identifying fusion circRNAs on simulated datasets, we then applied acfs to the APL dataset in which fusion circRNAs were experimentally validated^16^. For the seven APL samples (APL1, APL2, APL3, APL4, NB4s1, NB4s2, and NB4c) with known chromosomal translocation in the breakpoint cluster region (bcr), we identified three fusion junctions from PML to RARA and one fusion junction from RARA to PML (Fig. 4d). However, these fusion junctions suggested the existence of linear fusion transcripts but not fusion circRNAs. Similarly, we detected only one fusion junction between MLL and AF9 in both THP1 cells and APL0 sample. We also had negative results by running three other fusion-detecting tools (Table S2). Alternatively, we constructed a reference from the junction sequences suggested by Guarnerio *et al*., and directly aligned all sequencing reads against this reference. When requiring at least 4 nt overlap with the junction and allowing up to 4% errors in the reads, only fusion junctions of PML/RARA and MLL/AF9 were detected, whereas the counterpart fusion junctions of RARA/PML or AF9/MLL were not (Table S3). Taking both lines of evidence together, we concluded that we were unable to identify fusion circRNAs in this RNA-Seq dataset. However, it is likely that those experimentally validated circRNAs are of low abundance, and the sequencing depth was not deep enough for computational detection. To sum up, acfs allows efficient detection of fusion circRNAs and our reanalysis of the published data suggests the importance of unbiased profiling of samples of interest with sufficient sequencing depth.

## Discussion

As a group of much neglected noncoding RNA, circRNAs have been recently identified in many organisms from human to yeast with temporospatial-specific expression profiles^6^,^10^,^13^,^14^,^15^,^27^. Although tens of thousands of circRNAs have been identified to date, we are still far from being able to chart a comprehensive landscape for them. More importantly, investigation of both the expression dynamics and the underlying regulatory mechanism requires accurate abundance estimation. Many tools have been developed for circRNA identification and quantification using RNA-Seq data, yet their performances vary quite substantially on the following four aspects. First, many tools depend on a high-quality known gene annotation, which is absent for most non-model organisms and still far from comprehensive even for the human genome. On the contrary, *de novo* prediction tools are not hindered by this limitation and can identify circRNAs in an unbiased manner. Second, the predicted back-splice junctions (BSJs) should be recognizable to the splicing machinery. While a few tools check for the canonical 4-mer splicing motifs, the rest of the tools do not examine the authenticity of the predicted BSJs, which give rise to not only false negatives but also false positives. Third, the accuracy of abundance estimation is not satisfactorily high for most of the tools benchmarked. Last but not least, only a few tools support single-end data whilst most others can only work with paired-end data. In this study, we present a standalone pipeline acfs that addresses all the aforementioned challenges with balanced performance and computational requirements. Benchmarks using several simulated and real-world datasets clearly demonstrated high accuracy and low false discovery rate of acfs (Fig. 2, Table S1). Several factors, including the circRNA abundance and the sequencing read length, could influence the performance. Using simulation, we show that while the accuracy reaches a plateau around 5~10 reads overlapping with the BSJ, lowly expressed circRNAs are difficult to identify (Fig. S8, Table S4), suggesting the necessity of sufficient sequencing depth in real-world applications. Also as expected, longer sequencing length contributes to the improved ability to detect and quantify circRNAs (Fig. S8, Table S4). Applying acfs on RNA-Seq datasets from leukemia samples, we identified thousands of circRNAs and, among them, a set of differentially expressed circRNAs which could potentially serve as diagnostic biomarkers (Fig. 3f–h). Given their enrichment in the exosomes^22^ and the extraordinary long half-life^8^ presumably owing to the circular structure, it is foreseeable that circRNAs serve in many roles from transcriptome characterization to liquid biopsy and treatment monitoring.

CircRNAs can originate from gene loci that are consistent with the reference genome, as well as from fusion genes caused by chromosomal translocations. Chromosomal translocations have been observed in many hereditary diseases as well as cancers, and many characteristic fusion genes have been casually linked to the etiology of the diseases. Since most studies on circRNAs focus on cell lines and only a few on normal tissue samples, the complexity of circRNA landscape in disease samples remains largely unexplored, letting alone the appreciation of their pathological impact. Recently, a study for the first time experimentally validated both the existence and the functionality of a few circRNAs in leukemia^16^. Although many tools have been developed to identify either circRNAs or fusion genes, currently none could directly report fusion circRNAs. In order to facilitate further genome-wide profiling, acfs can identify and quantify the diagnostic fusion junctions of fusion circRNAs. Comparing with several state-of-the-art fusion gene identification tools on simulated datasets, acfs consistently recovered most of the true fusion circRNAs with the fewest false positives (Fig. 4b,c). Nevertheless, future experiments are needed for further characterization of fusion circRNAs and their functional interpretation.

Computational prediction of circRNA functions relies on the full-length sequences which is the starting point of the functional studies such as transcriptional regulation and RNA binding protein/miRNA decoying. More importantly, the knowledge of full-length sequences of circRNAs could enhance the abundance estimation as required in the realignment step in acfs. Due to the relative short sequencing read length in many studies, it is challenging to directly identify the full-length sequences of circRNAs in a genome-wide manner. Increasing the length of both the sequencing library and the reads could shed more light on the internal exon/sequence of circRNAs. Indeed, with 250 bp paired-end RNA-Seq data^18^ or *de novo* assembly^17^, thousands of novel circRNAs have been identified in two recent studies, many of which are characterized by alternative RNA processing event such as intron retention, exon skipping and alternative splicing. The observed complexity of circRNA isoforms is consistent to that of the linear transcript isoforms estimated using long read sequencing technologies^44^, which points clearly to the splicing machinery as the source and further emphasizes the importance of BSJ inspection as implemented in acfs. For circRNAs of length beyond the capacity of NGS platform, long read sequencing technologies, such as PacBio, could be applied to reveal their complete sequences^13^, albeit not permitting genome-wide application at the moment due to the cost and shallow throughput. Compiling a comprehensive catalog of circRNA isoforms remains an important task for future research.

To sum up, charting the landscape of circRNAs and their expression dynamics, including fusion circRNAs, could not only deepen our understanding of the complexity of the transcriptome but also shed light on the potential usage of circRNAs as a biomarker and even treatment agents. We believe our freely available tool acfs, featured with *de novo* BSJ identification, high accuracy, and low FDR, can be widely applied to a variety of circRNA studies, especially those involving non-model organisms and cancer samples.

## Methods

### Simulated Benchmark datasets

Fasta sequences and gtf annotation of human (hg19) RefSeq transcripts were downloaded from UCSC Genome Browser. Simulated single-end (SE) and paired-end (PE) linear reads were randomly generated from 52408 RefSeq transcripts of length longer than 300 nt. To simulate circRNAs with *bona fide* exon structures, we select RefSeq transcripts with at least three exons, and construct BSJs with one or two internal exons that are randomly selected. To reflect the observation that multiple circRNAs could originate from the same linear transcript, we simulated two circRNAs for a few RefSeq transcripts, reaching a total of 61488 artificial circRNAs. Sequences of circRNAs were derived by concatenating all exonic sequences between the selected exon(s), and those of length shorter than 100 nt were discarded. Simulated SE circRNAs reads were randomly generated spanning the BSJ. For PE circRNA reads, we first randomized an insert-size from a normal distribution N(200, 50), and then randomized a left-most position such that the resultant insert spanned the BSJ. To rule out the potential influence of the transcript abundance, we simulated 20 reads or read-pairs from each of the circRNAs and RefSeq transcripts. Fusion circRNAs were simulated by random selection of two internal exon regions from two RefSeq genes (A_[i,j]_, B_[m,n]_, where i< = j, and m< = n), and sequential joining of A_j_ to B_m_, and B_n_ to A_i_. We simulated 10 such fusion circRNA events and simulated 10 reads from each of the fusion junctions. All simulation scripts are available in the acfs package.

### Evaluation with leukemia patient data

APL patient RNA-Seq data were obtained from (Guarnerio *et al*.^16^). CN-AML patient RNA-Seq data were obtained from (Garzon *et al*.^31^). All samples were supplied to acfs and run with default parameters. To benchmark the efficacy of finding fusion junctions for fusion circRNAs, segemehl, MapSplice2 and Tophat-Fusion were also run with default/recommended parameters. As an independent approach to detect fusion circRNA, we constructed a database consisting of the fusion junction sequences suggested by the authors and aligned all sequencing reads to this reference with bowtie allowing up to 3 mismatches. Then we counted the number of reads that span the fusion junctions with at least 4 nt overhang to avoid ambiguous alignments.

### MiRNA-guided cleavage site on circRNA

To predict if a certain circRNA could be cleaved by miRNA-bounded AGO2 complex, we concatenated all the exonic sequences for each circRNA, and counted the number of full-length miRNA binding sites with less than 5 mismatches located beyond the seed region for all miRNAs (deposited in miRBase^45^ version 21).

### Estimation of folding energy for circRNA

RNAfold^46^ was used to estimate the secondary structure and folding energy for each candidate circRNA, with recommended parameters (-circ -MEA -d2 -p).

### Data Availability

Acfs is freely available at https://github.com/arthuryxt/acfs under GNU v3 license.

## Additional Information

**How to cite this article**: You, X. and Conrad, T. OF. Acfs: accurate circRNA identification and quantification from RNA-Seq data. *Sci. Rep.* **6**, 38820; doi: 10.1038/srep38820 (2016).

**Publisher's note:** Springer Nature remains neutral with regard to jurisdictional claims in published maps and institutional affiliations.

## Supplementary Material

Supplementary Materials

Supplementary Tables

## Figures and Tables

**Table 1:** Overview of circRNA detection methods.

Tool	De novo?	Check splice site	Realignment	Single-end?	Mapper	Version
acfs	yes	full	yes	yes	BWA-MEM	2.2
segemehl	yes	no	no	yes	segemehl	0.2.0
CIRCexplorer	no	GU-AG GC-AG AU-AC	no	yes	STAR	1.1.3
KNIFE	no	no	no	yes	Bowtie1 and 2	NA
MapSplice2	no	no	no	yes	Bowtie1	2.2.0
circRNA_finder	yes	GU-AG GC-AG AU-AC	no	no	STAR	NA
CIRI	yes	GU-AG	no	yes*	BWA-MEM	1.2
find_circ	yes	GU-AG	no	no	Bowtie2	1.0

^*^Although CIRI can take in SE reads, it never finishes within 10000 min in our simulations.
