# Supplementary material for: Acfs: accurate circRNA identification and quantification from RNA-Seq data
Source: Sci Rep. 2016 Dec 8;6:38820. doi: 10.1038/srep38820 (PMC5144000; doi:10.1038/srep38820)
Supplement: Supplementary Materials [file srep38820-s1.doc]

**Supplementary Materials**

**Acfs: accurate circRNA identification and quantification from RNA-Seq data**

Xintian You*^1^ and Tim OF Conrad^1,2^

1. Department of Numerical Mathematics, The Zuse Institute Berlin, Berlin, 14195, Germany

2. Institut für Mathematik, Freie Universität Berlin, Berlin, 14195, Germany

**Supplementary Notes**

During PE to SE conversion, there are three scenarios where a PE read overlap with a BSJ as illustrated in Fig S7.

1) only one read mate overlaps with the BSJ, and the other read mate locates in distance (Fig S7a). In this case, we can safely treat the two read mates as two independent SE reads with no information loss.

2) a PE read flanks the BSJ but neither mate directly overlaps with the BSJ (Fig S7b). Due to the uncertainty of the length of the insert, it is often difficult to tell the exact BSJ. Since acfs tries to pinpoint the exact genomic position of the BSJs, such kind of PE reads are not used by acfs to predict circRNAs.

3) both read mates overlap with the BSJ (Fig S7c). In this case, the two read mates share a piece of common sequence which can be used to merge the two into one sequence. The merged sequence is then treated as one SE read so that a PE read is counted only once during the quantification step.

Note that this conversion might be quite time consuming for large PE RNA-Seq datasets with long sequencing length. Since reads directly mappable to genome and/or transcriptome references do not contribute to the identification of circRNAs, we recommend to align the raw reads to genome and/or transcriptome references first and process the unmapped reads for acfs.

Parameters used for simulation benchmark (in below we listed the parameters that need to be specified during runtime):

| acfs | default |
| --- | --- |
| segemehl | default |
| CIRCexplorer | (parameters for STAR, as recommend in the manual) --readFilesIn SE.fa --chimSegmentMin 20 --chimScoreMin 1 --alignIntronMax 100000 --outFilterMismatchNmax 4 --alignTranscriptsPerReadNmax 100000 --outFilterMultimapNmax 2 |
| KNIFE | junction_overlap=15, ntrim=50 (as recommend in the manual) |
| MapSplice2 | --fusion-non-canonical --min-fusion-distance 100 (to match with the minimal length of artificial circRNA from which we simulate reads) |
| circRNA_finder | default |
| CIRI | -T 19 (as recommend in the manual) |
| find_circ | default |

**Supplementary figure legends**

**Figure S1 | Cumulative distribution of strength of annotated splicing sites in human transcriptome.** Using a threshold of 10 as default in acfs, 95% of the annotated splicing sites in RefSeq human database can be detected.

**Figure S2 | Length distribution of genomic oligo(A) sequences in human transcriptome.** CircRNAs could be captured during the poly(A) selection if they contain a long stretch of internal A(s) although they don’t possess poly(A) tails. The length distribution shows that circRNAs should be mostly depleted by poly(A) selection.

**Figure S3 | Cumulative distribution of the splice distance for circRNAs predicted in Dataset A (zoom-in).** Over 20% of the circRNAs predicted by segemehl has proximal splice sites within 60bp.

**Figure S4 | Potential reasons for a subset of circRNAs with weak RNase R resistance.** (a) Higher miRNA binding potential. Predicted circRNAs were separated into seven groups by the minimum number of mismatches in the miRNA binding sites (X-axis). To be qualiried as a miRNA binding site, no mismatch is allowed in the miRNA-seed-matching region. The value on Y-axis marks the RNase R resistance of circRNAs (RNase R treated vs mock-treated, log2 transformed). CircRNAs with more mismatches in the miRNA-binding sites (and therefore lower binding affinity) have, in general, higher resistance. Pairwise t-test p-values were showed in the top. (Unpaired one-sided t-test with Welch correction for the unequal variance) (b) Less complex RNA secondary structure. The length-normalized minimum free energy (mfe) is shown on Y-axis. Although the overall correlation is weak, it is clear that the RNase R sensitive circRNAs (top-left corner) have lower normalized mfe as predicted by their simpler secondary structures.

**Figure S5 | Expression of linear transcript does not separate APL and CN-AML samples as good as that of circRNAs.** (a) Pairwise correlation of the expression of all linear protein-coding genes among five APL and five CN-AML samples. (b) Pairwise correlation of the expression of all circRNAs. (c) The expression of those 80 circRNA-hosting genes does not separate APL from CN-AML samples (related to Fig 3f).

**Figure S6 | CircRNAs whose hosting genes are frequently mutated in AML samples are higher expressed.** CircRNAs were grouped by whether or not their hosting genes are reported to be frequently mutated in AML samples. CircRNAs with frequently mutated hosting genes are of significant higher abundance compared to those without (p-value = 1.378e-05, unpaired one-sided t-test with Welch correction for the unequal variance).

**Figure S7 | Scenarios of PE-to-SE conversion**. Description see Supplementary Note.

**Figure S8 | Effect of circRNA abundance and read length on the performance of acfs.** F1 accuracy for circRNA identification (a) and expression quantification (b) in a “dope-in” simulation with various number of reads per simulated circRNA (X-axis). The F1 accuracy is relatively poor when there are only one or two reads due to the high probability that all reads span the BSJ for few nucleotides and therefore cannot be confidently mapped. As the number of reads increases, the probability that all reads cannot be confidently mapped decreases exponentially and the F1 accuracy would increase and reach a plateau. Also as expected, longer sequencing length contributes to the improved ability to detect and quantify circRNAs. The FDR for identification (c) and quantification (d) is consistently low (below 0.001) in all cases.

**Supplementary Tables** (In a separated file)

Table S1. Benchmark summary (related to Fig 2).

Table S2. Number of fusion junctions detected in the APL sample using acfs, TophatFusion, segemehl, and MapSplice2 (related to Fig 4).

Table S3: Mapping all reads directly to fusion junctions support characteristic linear fusion transcripts but no fusion circRNAs.

Table S4: Effect of circRNA abundance and read length on the performance of acfs (related to Fig S8).

Figure S1

Figure S2

Figure S3

Figure S4

Figure S5

Figure S6

Figure S7

Figure S8
